# Supplementary material for: Quantifying and Cataloguing Unknown Sequences within Human Microbiomes
Source: mSystems. 2022 Mar 8;7(2):e01468-21. doi: 10.1128/msystems.01468-21 (PMC9052204; doi:10.1128/msystems.01468-21)
Supplement: TEXT S1 [file msystems.01468-21-s0001.pdf]

Supplementary text

CheckV predicted viral contigs

All UCs were processed through the CheckV pipeline [1] to identify the UCs that were likely to belong to viruses. A total of 47,702 UCs were predicted to be of viral origin and 7,696 of them were at least 1kb long. A set of 11,121 of these UCs were predicted to have at least one viral gene and 1,712 UCs of this subset were at least 1kb long. These 1,712 UCs were mapped against the results of the most recently carried out BLASTX analysis for validation. 529 of the predicted viral contigs matched to bacterial protein sequences with low sequence identity with a mean percent identity of 48.43. However, these results are based on short protein sequence hits on bacterial proteins indicating that these UCs are likely to be phage genomic signatures that matched bacterial protein in absence of a phage sequence in the database specifically as protein-based similarity searches are able to identify distantly related homologues of query sequences. These results can help to stipulate that the actual diversity of virus sequences present in the UCs set is largely underestimated. It is highly likely that a range of contigs that match distantly related protein sequences of bacterial origin are in fact derived from unknown and uncultured novel viruses, such as phages, that infect bacteria.

The large unknown contigs

The largest multi metagenome UCs (figure S5(a)), was assembled from SRR2037089 from the oral metagenome and was 14,958 bases long. It was clustered with 33 other contig sequence assembled from 12 distinct samples from oral (n=8; PRJNA230363), sputum (n=3; PRJEB10919) and saliva (n=23; PRJEB14383) microbiomes. These three distinct studies contained samples from distinct geographic locations: PRJNA230363 from China, PRJEB14383 from the Philippines and PRJEB10919 from South Africa suggesting that this unknown organism is broadly distributed in its association with humans. The second-largest member of this cluster was 9,791 bases long and was assembled from a separate sample (SRR2037087) from the same study. This large contig was deemed to be identical to the cluster representative. The largest cluster member from the saliva microbiome was 1.5kb long and was assembled from ERR1474566. On the contrary,

the contigs assembled from the sputum microbiomes were significantly smaller with lengths ranging between 479-533 bases, indicating the fragmented assembly and the presence of partial sequences.

A large contig of length 21,357 was identified in the oral microbiome shown in figure S5(b). This contig was assembled from run ERR1611386 and was clustered with 16 other sequences from BioProjects PRJEB12831 and PRJEB15334. Other members of the clusters originated from 5 distinct samples and were between 306-6,109 bases long. This contig contained the largest predicted ORF that was 6,898 residues long. 14 out of the 16 other contigs within the cluster contained partial sequences belonging to this ORF. This contig also did not have a taxonomic homologue identified in any of the most recent similarity sequence-based searches. Additionally, the largest ORF was predicted to contain P-loop containing nucleoside triphosphate hydrolases (SUPERFAMILY: SSF52540) signatures.

The largest cluster contained 153 sequences (figure S5(c)) had a cluster representative that was 6,642 bases long assembled from sample ERR1297807 from PRJEB12357. This cluster representative was predicted to contain 9 distinct ORFs. The cluster contained 35 other sequences that were at least 1kb long. Additionally, other contigs (6,015 bases long from ERR537012 and 5,344 bases long from ERR537011) from as a separate study (PRJEB6542) were found in this large cluster.

References

[1] Stephen Nayfach, Antonio Pedro Camargo, Frederik Schulz, Emiley Eloe-Fadrosh, Simon Roux, and Nikos C. Kyrpides. “CheckV assesses the quality and completeness of metagenome-assembled viral genomes”. In: *Nature Biotechnology 2020 39:5* 39.5 (2020), pp. 578–585. DOI: 10.1038/s41587-020-00774-7.
